# Image reconstruction of mMR PET data using the open source software STIR

**DOI:** 10.1186/2197-7364-1-S1-A44

**Published:** 2014-07-29

**Authors:** Pawel Markiewicz, Kris Thielemans, Ninon Burgos, Richard Manber, Jieqing Jiao, Anna Barnes, David Atkinson, Simon R Arridge, Brian F Hutton, Sébastien Ourselin

**Affiliations:** Centre for Medical Image Computing, University College London, London, UK; Institute of Nuclear Medicine, University College London, London, UK; Dementia Research Centre, University College London, London, UK; Centre for Medical Imaging, University College London, London, UK

Simultaneous PET and MR acquisitions have now become possible with the new hybrid Biograph Molecular MR (mMR) scanner from Siemens. The purpose of this work is to create a platform for mMR 3D and 4D PET image reconstruction which would be freely accessible to the community as well as fully adjustable in order to obtain optimal images for a given research task in PET imaging. The proposed platform is envisaged to prove useful in developing novel and robust image bio-markers which could then be adapted for use on the mMR scanner.

STIR (Software for Tomographic Image Reconstruction [1]), an open source C++ library, has been used as a basis for this platform. However, a number of practical issues have to be addressed before useful reconstructed images can be obtained. Many of the practical issues have been addressed and reconstructions of two datasets (phantom and human brain) are demonstrated.

The reconstruction pipeline involves the following:

- Conversion of the Siemens Dicom files to the STIR interfile format.
- Histogramming the emission data.
- Component-based normalisation.
- Estimation and correction for attenuation and scatter using atlas based *µ*-maps [2].
- Correction for randoms.
- Iterative image reconstruction ([3–5]).

**Figure 1:**
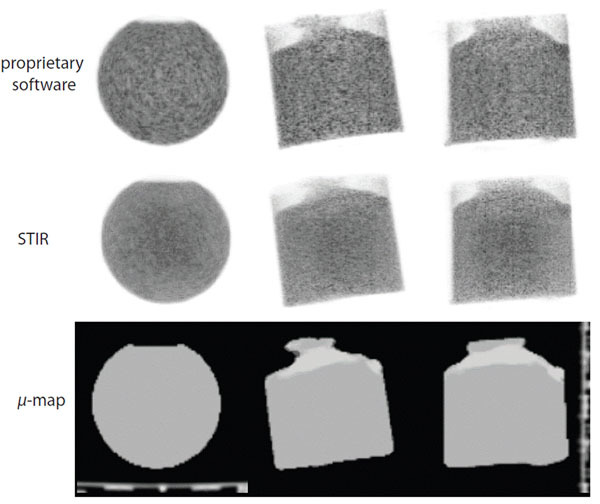
Image reconstruction of the phantom using Siemens proprietary software (top) and STIR (middle). Bottom: the MRI derived *µ*-map.

Two datasets were used: uniform cylinder phantom and FDG-PET human brain scan. Figure 1 shows the OSEM reconstruction using the of-line version of the Siemens Healthcare reconstruction software which was made available for this project (top); the STIR reconstruction (middle); and the *µ*-map including the bed component (bottom).

**Figure 2:**
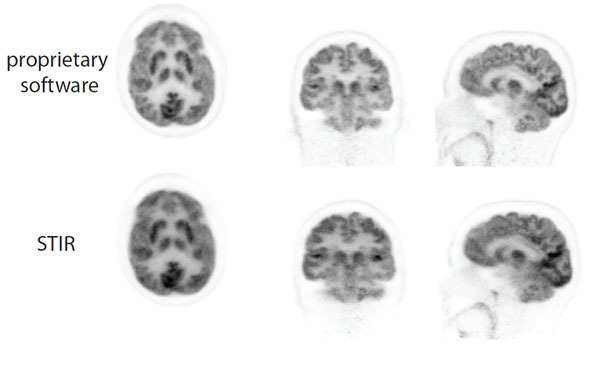
Image reconstruction of the human brain FDG-PET dataset using Siemens proprietary software (top) and STIR (bottom).

The reconstruction of the FDG human brain dataset is presented in Figure 2 using Siemens proprietary software (top) and the proposed STIR pipeline (bottom).

It was demonstrated that STIR image reconstruction of PET mMR data is possible paving the way to more advanced models included in the pipeline of 4D PET reconstruction.
